# First description of the male of *Hitobia makotoi* Kamura, 2011 (Araneae, Gnaphosidae)

**DOI:** 10.3897/zookeys.579.7489

**Published:** 2016-04-11

**Authors:** Bing Zhou, Haiqiang Yin, Xiang Xu

**Affiliations:** 1 College of Life Sciences, Hunan Normal University, Changsha 410081, China

**Keywords:** East Asia, ground spider, taxonomy

## Abstract

The male of *Hitobia makotoi* Kamura, 2011 is described for the first time from Hengshan Mountain, Hunan Province, China. This species was originally described from Amami-ôshim Island, Japan and has been recorded from Gaoligong Mountain, Yunnan Province, China. Morphological descriptions and illustrations of both sexes of this species are given.

## Introduction

The spider genus *Hitobia* is distributed in East Asia with 16 species described to date, half of which are described by only the male or only the female (World Spider Catalog Version 2016, Wang and Peng 2014, Kamura 2011).

The species *Hitobia makotoi* Kamura, 2011 was first described based on female specimens only from Amami-ôshim Island, Japan. Wang and Peng (2014) identified a female specimen from Gaoligong Mountain, Yunnan Province, China as this species, although the spermathecae of this specimen are slightly different from those of the holotype in the original description of Kamura (2011). Recently, during examination of the spider specimens collected from Hunan Province, one female and four males were found, which can be identified as *Hitobia makotoi* based on comparison with the type specimens. The female is redescribed and the male is described for the first time in the present paper.

## Material and methods

Specimens were examined with an Olympus SZX16 stereomicroscope. Details were further studied with an Olympus BX53 compound microscope. The illustrations were made using an Olympus drawing tube and inked on ink jet plotter paper. Photos were taken with a Canon PowerShot G12 digital camera mounted on an Olympus BX53 compound microscope and compound focus images were generated using Helicon Focus software (3.10 Free). Both the male palp and the female epigynum were detached from the spiders’ bodies for examination and illustration.

All specimens are deposited in the College of Life Sciences, Hunan Normal University (HNU).

All measurements are given in millimeters. Leg measurements are given as: total length (femur, patella + tibia, metatarsus, tarsus). The following abbreviations are used in the text:

ALE
 anterior lateral eye 


AME
 anterior median eye 


MOA
 median ocular area 


PLE
 posterior lateral eye 


PME
 posterior median eye 


## Taxonomy

### Family Gnaphosidae Pocock, 1898
*Hitobia* Kamura, 1992

#### Hitobia makotoi

Taxon classificationAnimaliaAraneaeGnaphosidae

Kamura, 2011

Figs 1 , 2 , 3 , 4

Hitobia makotoi Kamura, 2011: 104, figs 3–7 (description and illustration of female).
Hitobia makotoi : Wang and Peng 2014: 31, figs 17–23 (description and illustration of female).

##### Material examined.

2 ♂ (HNU), Cangjingdian (27°16.14'N, 112°41.72'E, 950 m), Hengshan Mountain, Hengyang City, **Hunan Province, China**, 8 July 2014; 1 ♂, 1 ♀ (HNU), Lingzhiquan (27°16.28'N, 112°42.13'E, 650 m), Hengshan Mountain, Hengyang City, **Hunan Province, China**, 8 July 2014; 1 ♂ (HNU), Shumuyuan (27°15.93'N, 112°43.34'E, 360 m), Hengshan Mountain Hengyang City, **Hunan Province, China**, 10 July 2014. All specimens were collected by Bing Zhou, Cheng Wang, Jiahui Gan and Yuhui Gong.

##### Diagnosis.

**Figure 1.**
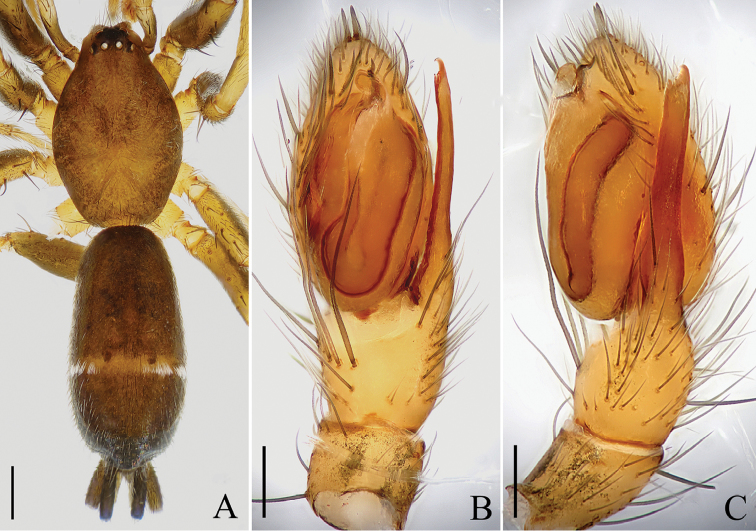
Male of *Hitobia makotoi* Kamura, 2011, based on the specimen from Hengshan. **A** Habitus, dorsal view **B** Left palp, ventral view **C** Same, retrolateral view. Scale bars: 0.5 mm (**A**); 0.1 mm (**B, C**).

**Figure 2.**
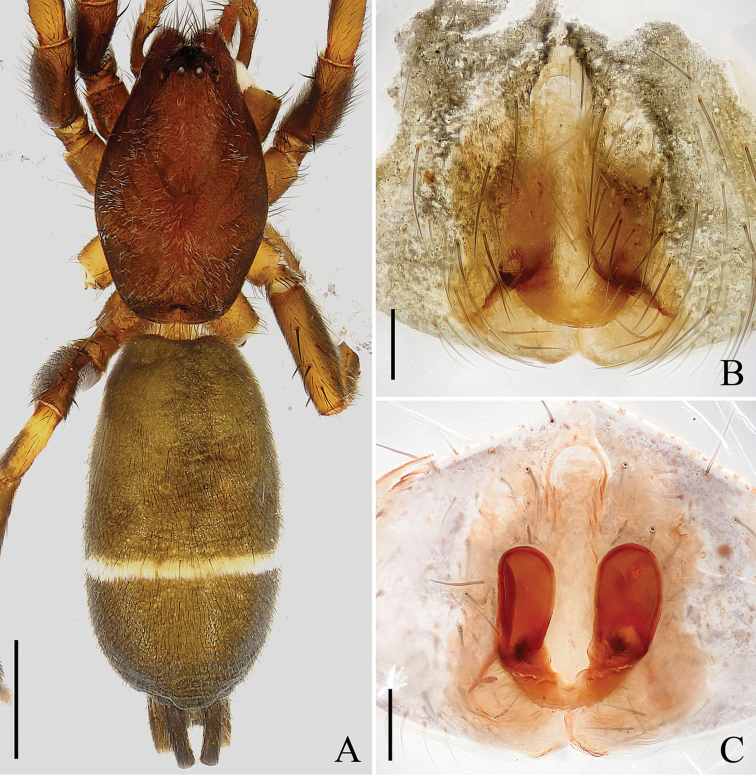
Female of *Hitobia makotoi* Kamura, 2011, based on the specimen from Hengshan. **A** Habitus, dorsal view **B** Epigynum, ventral view **C** Vulva, dorsal view. Scale bars: 1 mm (**A**); 0.1 mm (**B, C**).

**Figure 3.**
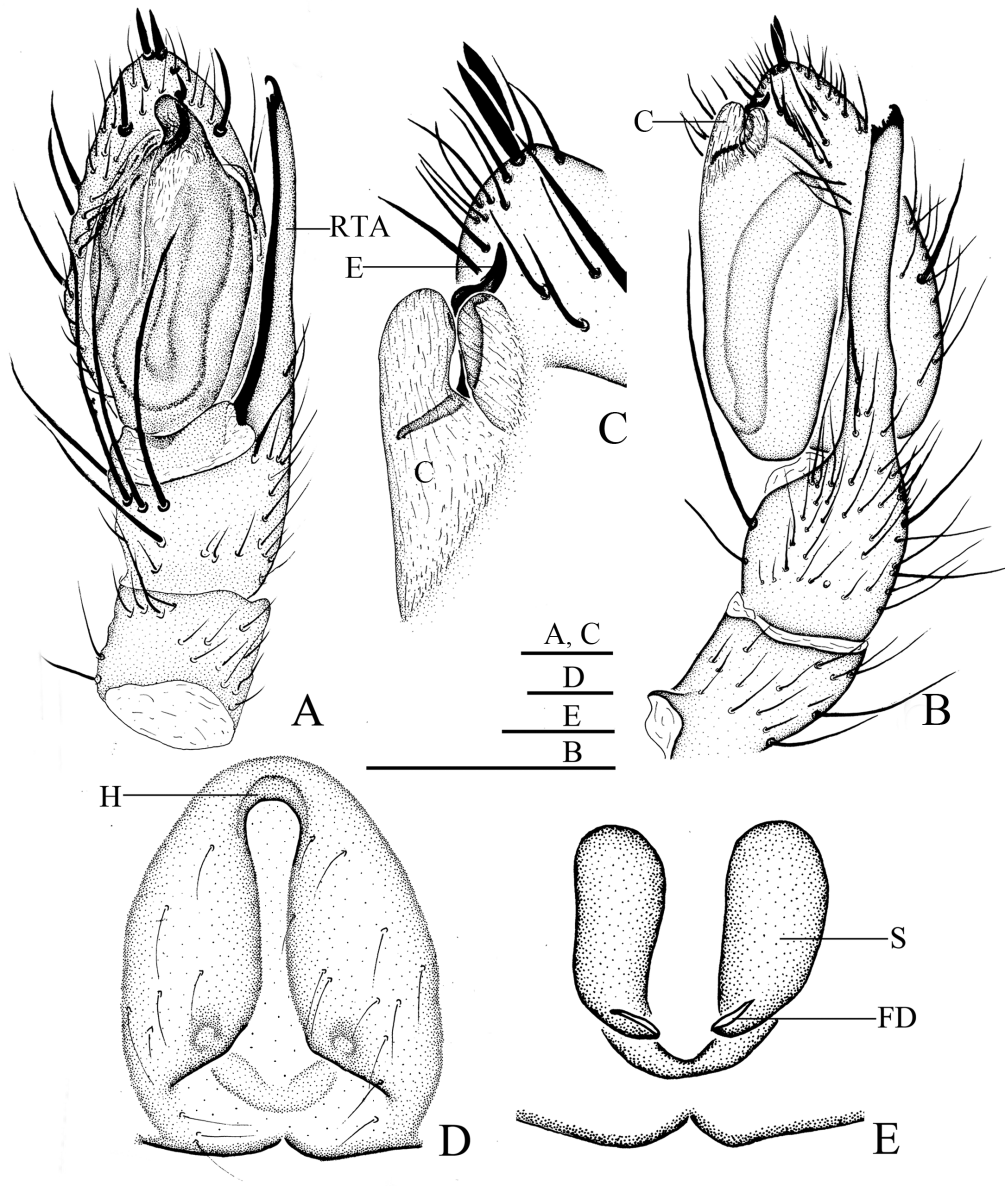
*Hitobia makotoi* Kamura, 2011. **A–C** Male: **A** Left palp, ventral view **B** Same, retrolateral view **C** Enlarged embolus and conductor, retrolateral view **B, C** Female: **B** Epigynum, ventral view **C** Vulva, dorsal view. Scale bars = 0.1 mm. Abbreviations: C - conductor, E - embolus, FD - fertilization duct, H - hood, RTA - retrolateral tibial apophysis, S - spermatheca.

Male of *Hitobia makotoi* can be distinguished from all other *Hitobia* by the extraordinarily elongated retrolateral tibial apophysis which is nearly as long as the cymbium, and its distal end serrated and with a small hook apically (Figs 1B, C; 3A, B). The female of *Hitobia makotoi* is similar to that of *Hitobia unifascigera* (Bösenberg & Strand, 1906) in having a transverse white band on the posterior part of opisthosoma, but can be distinguished from the latter by the following characters: epigynal hood situated on the anterior part of epigynum, but situated at the middle part in *Hitobia unifascigera*; atrium vertically elongated, and almost as long as the epigynum, but short, and half as long as the epigynum in *Hitobia unifascigera* (Figs 2B, 3D, cf. fig. 633e in Yin et al. 2012 and fig. 89H in Song, Zhu and Zhang 2004); and finally, a long and thin spermathecae in *Hitobia makotoi*, but thick and massive in *Hitobia unifascigera* (Figs 2C, 3E, cf. fig. 633f in Yin et al. 2012 and fig. 89I in Song, Zhu and Zhang 2004).

##### Description.

**Male**: Body length 3.92. Prosoma length 1.78, width 1.18; opisthosoma length 2.18, width 1.01. Clypeus height 0.04. Carapace black brown, oval, widest at coxae II and III. Fovea and cervical grooves indistinct (Fig. 1A). Eye measurements and interdistances: AME 0.08, ALE 0.10, PME 0.08, PLE 0.10, AME–AME 0.03, AME–ALE 0.01, PME–PME 0.08, PME–PLE 0.05, ALE–PLE 0.07. MOA anterior width 0.15, posterior width 0.20, length 0.07. Both eye rows recurved. Chelicerae with three promarginal (the middle one largest) and one retromarginal teeth. Endites yellowish, with some soft short hairs on the inner side. Labium yellowish brown, longer than wide. Sternum decorated with many black spots, anterior straight and posterior subacute. Legs femora I–IV blackish brown, but yellowish brown dorsally. Trochanters I and II without ventral notch, III and IV each with a shallow ventral notch. Leg spination: femora: I, II, IV d1-1-1, p1-0-0, III d1-1-1, p1-1-0, v1-1-0; patellae: III, IV r0-1-0; tibiae: I p1-0-0, v2-2-2, II p1-0-1, v2-2-1, III d2-2-0, p0-0-1, v2-2-1, r0-1-1, IV d1-0-0, p1-1-1, v1-0-1, r1-0-1; metatarsi: I v2-0-0, II p0-1-0, v2-0-0, III d0-1-1, p1-1-1, v0-0-1, r1-1-2. Leg measurements: I 3.20 (0.88, 1.28, 0.60, 0.44), II 3.48 (1.00, 1.32, 0.68, 0.48), III 3.00 (0.84, 0.96, 0.76, 0.44), IV 4.12 (1.16, 1.36, 1.04, 0.56). Leg formula: IV-II-I-III. Opisthosoma long and oval, covered all over with villi, and with a transverse white stripe postero-dorsally; venter light brown. Spinnerets cylindrical and blackish brown.

Male palp (Figs 1B, C, 3A–C): Tibia short, with several long prolateral macrosetae, retrolateral tibial apophysis extraordinarily elongated, nearly as long as cymbium, and its distal end serrated and with a small hook apically. Bulb long and oval, simple. Conductor membranous, relatively large, originating from the middle part of bulb, covering the base of embolus. Embolus short, twisted, originating from the prolateral top of bulb, mostly hidden under conductor and only its tip visible in ventral view. A small membranous process originating from the retrolateral top of bulb, protecting the embolus together with the conductor (Fig. 3C). Two strong macrosetae situated at the top of cymbium.

**Female**: Body length 5.45. Prosoma length 2.30, width 1.46; opisthosoma length 3.05, width 1.61. Clypeus height 0.06. Eye measurements and interdistances: AME 0.08, ALE 0.10, PME 0.08, PLE 0.10, AME–AME 0.03, AME–ALE 0.01, PME–PME 0.08, PME–PLE 0.06, ALE–PLE 0.10. MOA anterior width 0.18, posterior width 0.23, length 0.12. Leg spination: femora: I, II, IV d1-1-1, p1-0-0, III d1-1-1, p1-1-0, v1-1-0; patellae: III, IV r0-1-0; tibiae: I p1-0-0, v2-2-2, II p1-0-1, v2-2-1, III d2-2-0, p0-0-1, v2-2-1, r0-1-1, IV d1-0-0, p1-1-1, v1-0-1, r1-0-1; metatarsi: I v2-0-0, II p0-1-0, v2-0-0, III d0-1-1, p1-1-1, v0-0-1, r1-1-2. Leg measurements: I 3.80(1.28, 1.40, 0.68, 0.44), II 3.88 (1.24, 1.44, 0.68, 0.52), III 3.52(1.12, 1.16, 0.76, 0.48), IV 5.28(1.52, 1.80, 1.20, 0.76). Leg formula: IV-II-I-III.

Epigynum longer than wide, with a distinct anterior hood; atrium vertically elongated, and almost as long as epigynum, with the basal part very wide, and abruptly becoming narrow and extending to the anterior part (Figs 2B, 3D); spermathecae reniform, vertically elongated, separated from each other (Figs 2C, 3E).

##### Remark.

There are very small differences between the holotype female and the newly collected female specimen in the present study: the distal part of atrium is narrower and the basal part wider in the newly collected female specimen (Figs 2B, 3D) than in the holotype (Fig. 6 in Kamura 2011).

##### Distribution.

China (Hunan, Yunnan), Japan (Amami-ôshima Is.).

**Figure 4.**
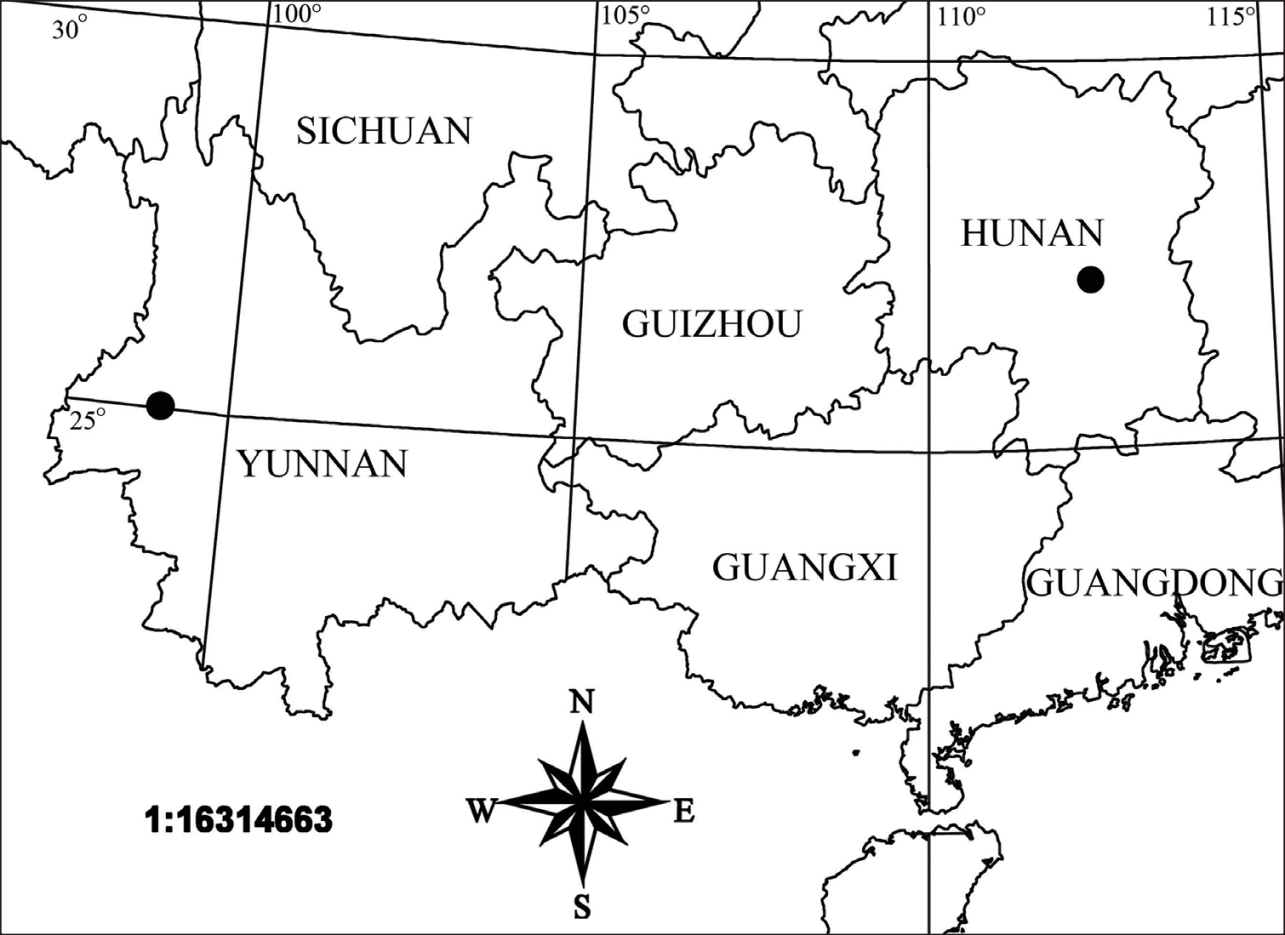
Distribution records of *Hitobia makotoi* Kamura, 2011 in China.

## Supplementary Material

XML Treatment for Hitobia makotoi
